# Water-processable PSS-free PEDOT:NO_3_/PVA colloid for sustainable organic electrochemical transistors

**DOI:** 10.1039/d6ma00785f

**Published:** 2026-08-14

**Authors:** Donia Saadi, Islam M. Minisy, Munise Cobet, Katharina Matura, Niko Münzenrieder, Markus Clark Scharber, Patrycja Bober, Niyazi Serdar Sariciftci, Serpil Tekoglu

**Affiliations:** a Faculty of Engineering, Free University of Bozen-Bolzano Bozen-Bolzano 39100 Italy donia.saadi@unibz.it; b Linz Institute for Organic Solar Cells (LIOS) and Institute of Physical Chemistry, Johannes Kepler University Linz Linz Austria serpil.tekoglu@jku.at; c Institute of Macromolecular Chemistry, Czech Academy of Sciences 16200 Prague Czech Republic

## Abstract

Organic electrochemical transistors (OECTs) enable low-voltage amplification of ionic signals directly in aqueous media, making them potential platforms for bioelectronic interfaces, neuromorphic circuits, and biosensors. Despite significant progress, most high-performance OECTs still rely on PEDOT:PSS, where the acidic, PSS-rich formulation and large excess of insulating polyanion can limit electronic transport and raise concerns regarding long-term stability and biocompatibility. In this work, we build on our previously reported PSS-free PEDOT colloids sterically stabilized by poly(vinyl alcohol) (PVA) and investigate this water-processable dispersion as an organic mixed ionic–electronic conductor (OMIEC) channel material for OECTs. The colloid, synthesized *via* oxidative polymerization of EDOT in water in the presence of neutral polymer PVA, yields highly stable dispersions that form smooth, conductive thin films suitable for device fabrication. Implemented as OECT channels, these PSS-free PEDOT:NO_3_/PVA films exhibit reproducible mixed ionic–electronic transport under aqueous operation with maximum transconductance values exceeding 10 mS. To the best of our knowledge, this is the first demonstration that a PVA-stabilized, PSS-free PEDOT colloid processed entirely from water can operate as an active OECT channel material, establishing a versatile route to neutral-polymer-stabilized, water-processable OMIECs for future soft and sustainable bioelectronic applications.

## Abstract 2

Water-processable PSS-free PEDOT colloid stabilized by PVA enables efficient mixed ionic–electronic conduction in OECTs with transconductance exceeding 10 mS.

## Graphical abstract

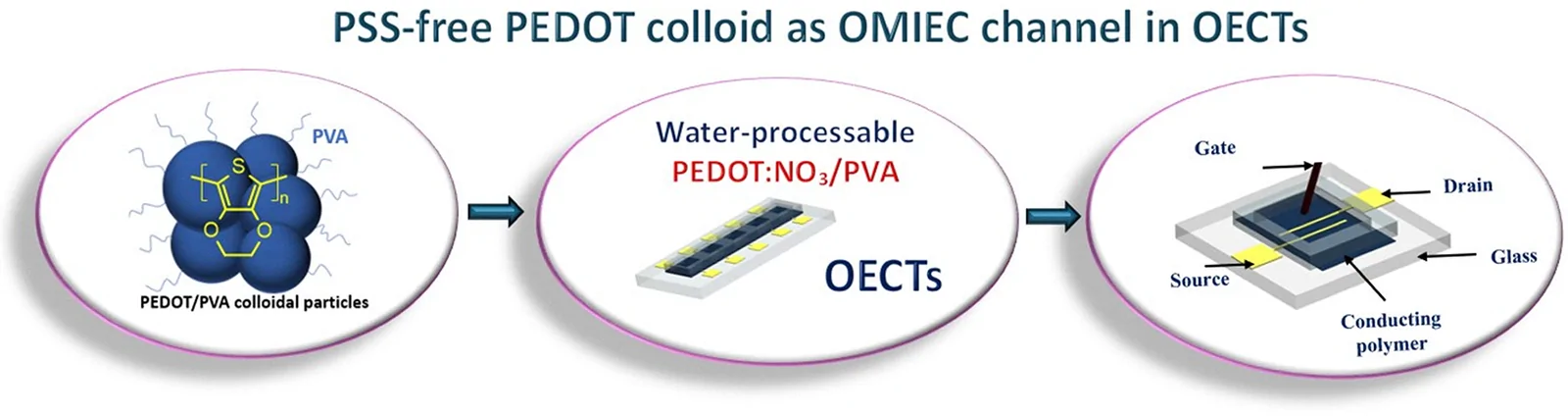

## 1. Introduction

Organic mixed ionic–electronic conductors (OMIECs) represent an emerging class of organic semiconductors that can transport both electronic charge carriers and ions simultaneously, enabling direct coupling to ionic signaling and its translation into electronic signals through ionic–electronic coupling phenomena.^1–5^ This dual transport mechanism allows efficient transduction of biological and chemical signals into electronic outputs, making OMIECs particularly attractive for bioelectronic interfaces. In addition to their mixed conduction, OMIECs offer intrinsic advantages such as mechanical softness, biocompatibility, chemical tunability, and solution processability, along with stable operation in aqueous environments, which collectively make them key active materials for next generation biosensing, bioelectronic and biomedical technologies.^1,2^

Among OMIEC-based devices, organic electrochemical transistors (OECTs), have emerged as a key platform for biosensing, biointerfacing, and neuromorphic systems.^6–14^ OECTs are three-terminal devices in which an OMIEC thin film forms the active channel between source and drain electrodes, and is in direct contact with a liquid or gel electrolyte that also interfaces the gate electrode.^3,15^ Upon application of a gate bias, ions from the electrolyte are driven into or out of the bulk of the OMIEC channel, inducing reversible electrochemical doping/de-doping that modulates the channel source–drain current.^12,16^ In contrast to field-effect transistors, where charge modulation is largely confined to an interfacial accumulation layer, OECTs operate through volumetric ionic–electronic coupling within the entire channel. This mechanism leads to high volumetric capacitance and large transconductance at low operating voltages, enabling efficient amplification of small ionic perturbations into measurable electronic signals in aqueous media. These characteristics make OECTs particularly well suited for applications involving chemical and biological sensing, signal recording, biomimetic devices in flexible or implantable formats.^12,16–18^

Poly(3,4-ethylenedioxythiophene):poly(styrenesulfonate) (PEDOT:PSS) is widely regarded as the benchmark p-type OMIEC for OECTs due to its high electrical conductivity, mixed ionic–electronic transport, aqueous dispersibility, and mechanical flexibility.^19,20^ In this polyelectrolyte complex, PEDOT is a positively charged π-conjugated polymer, while the polyanion PSS provides negatively charged sulfonate groups that both balance the positive charges on the PEDOT chains and act as a dispersing matrix,^19–22^ enabling the formation of stable aqueous dispersions and thin, homogeneous films by solution-based methods such as spin-coating, drop-casting and printing.^3,15,23,24^ The resulting water-based processability has made PEDOT:PSS a standard material for eco-friendly fabrication of bioelectronic devices.^25^ However, the sulfonic acid groups on PSS bear labile protons that make PEDOT:PSS formulations strongly acidic (typically pH 1.5–2.5),^26^ which has been linked to degradation of electrodes and active layers in semiconductor devices as well as to cytotoxic effects and sub-optimal cell adhesion in biointerfaces.^27^ Moreover, the acidic nature of PSS might pose challenges for long-term biocompatibility and stability.^27,28^ Therefore, OMIEC materials that simultaneously combine water-based processability, high mixed ionic–electronic conductivity, long-term stability, and improved biocompatibility without relying on excess acidic PSS or extensive post-treatment strategies, remains a key challenge and have recently driven increasing interest in the exploration of PSS-free PEDOT systems stabilized by alternative polymer matrices. Approaches in which PEDOT is stabilized by deoxyribonucleic acid (DNA), polysaccharides (cellulose nanocrystals, Sacran), and lignosulfonate as eco-friendly, bio-derived polyanions, have demonstrated promising routes to eliminate or reduce reliance on PSS while maintaining mixed ionic–electronic conduction.^29–32^ As previously reported, such biocompatible PEDOT–biopolymer composites have been used as channel polymers in biodegradable OECTs for sustainable bioelectronics.^31,32^ In parallel, electropolymerization strategies have been revisited to produce additive-free PEDOT films or PEDOT composites doped exclusively with small anions to avoid PSS while maintaining robust OECT operation.^3,20,23,31^

Building on this advances, we have shown in our previous work that PSS-free PEDOT colloids can be synthesized in water by using neutral, water-soluble polymers such as poly(vinyl alcohol) (PVA) as steric stabilizer during the oxidative polymerization of EDOT.^23^ Poly(vinyl alcohol) (PVA) is a hydrophilic, water-soluble polymer, widely used in organic and bioelectronic systems due to its excellent film-forming ability, flexibility and adhesion-enhancing properties.^22,33,34^ Additionally, PVA has attracted renewed interest as an environmentally friendly synthetic polymer and is regarded as one of the few fully synthetic carbon-chain polymers capable of biodegradation under both aerobic and anaerobic conditions.^35–37^ Therefore, PVA has attracted growing attention in OECT research. In earlier studies, PVA has been employed as solid electrolyte and in self-healing layers in OECTs.^14^ In a recent work by Gregorio *et al.*, mixed ionic–electronic conducting PVA/PEDOT:PSS hydrogels, were used directly as OECT channel materials, exhibiting maximum transconductance *g*_m_ value of about 1.05 µS.^38^ Ma *et al*. introduced a 3D-printable PEDOT:PSS–PVA ink, where PVA mainly acts as a mechanical and processing modifier, enabling fully 3D-printed OECTs with high transconductance ∼5 mS.^22^ In PEDOT:PSS/PVA composite based-OECTs developed by Kim *et al.*, PVA behaves as a neutral polymer matrix and crosslinker within the PEDOT:PSS network, exhibiting *g*_m_ values in the ≈0.3–1.5 mS range.^39^ However, its role as a neutral steric stabilizer enabling PSS-free PEDOT colloids for OMIEC channel applications remains largely unexplored.

**Fig. 1:**
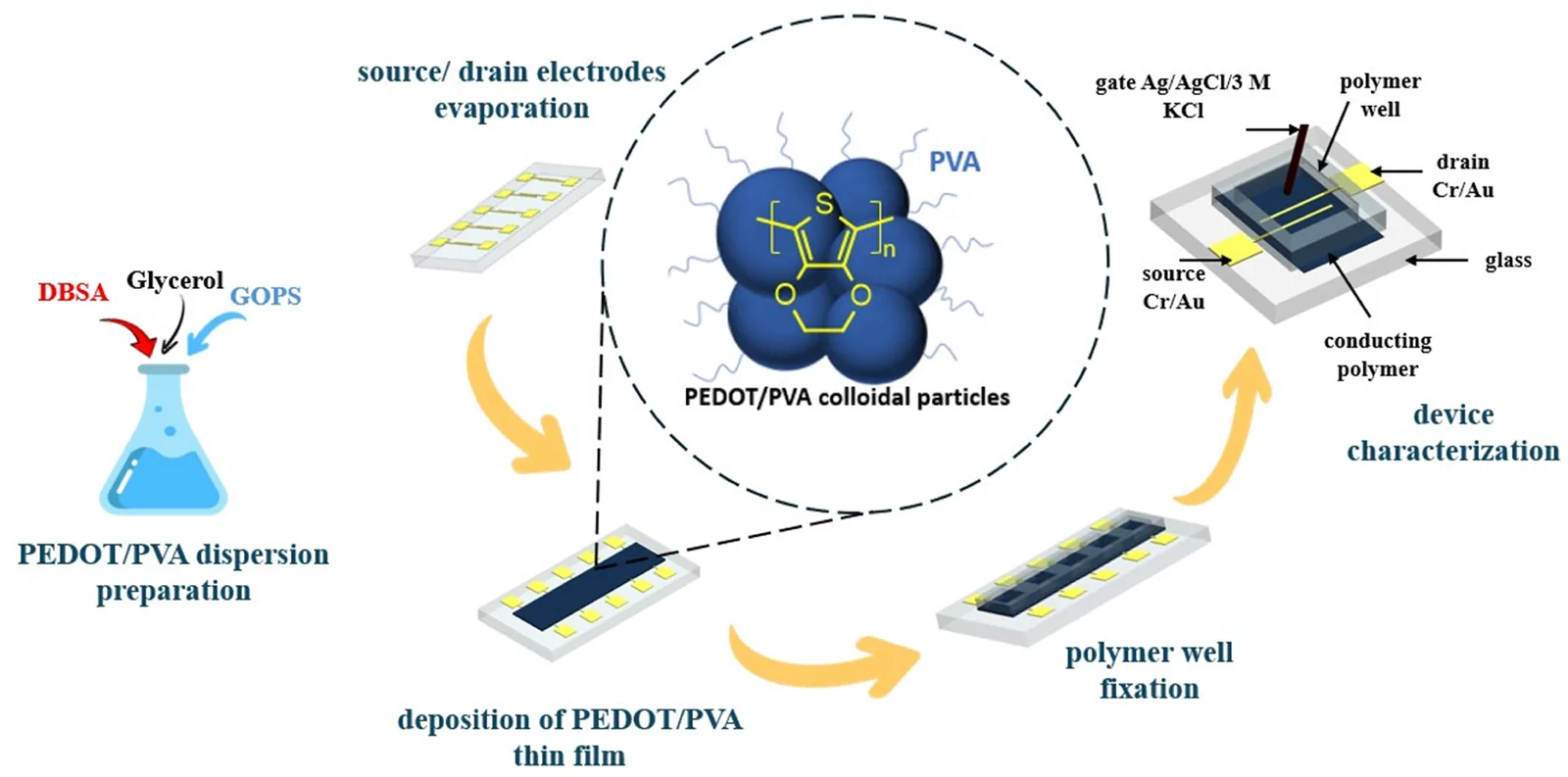
Schematic illustration of the fabrication of OECTs based on PEDOT:NO_3_/PVA dispersion.

In this study, we demonstrate PSS-free PEDOT colloids sterically stabilized by PVA as a water-processable OMIEC channel material for OECTs. The overall process is schematically illustrated in Fig. 1. The resulting OECTs exhibit reproducible mixed ionic–electronic transport under aqueous operation and transconductance values that are competitive with previously reported PVA-based OECTs, which have predominantly relied on PEDOT:PSS containing composites. At the same time, our approach avoids excess acidic PSS and uses PVA solely as a neutral steric stabilizer in a fully water-processable PEDOT dispersion. To the best of our knowledge, this is the first report of PSS-free PEDOT:NO_3_/PVA colloids functioning as OECT channel materials. The results position neutral-polymer-stabilized, PSS-free PEDOT:NO_3_/PVA colloids as a promising complement to existing PVA-based OECTs architectures for soft and sustainable bioelectronics. Although the present PEDOT:NO_3_/PVA formulation is not expected to be fully biodegradable due to the presence of PEDOT, its entirely water-based processing, replacement of the strongly acidic PSS polyelectrolyte with a neutral PVA steric stabilizer, and incorporation of a polymer matrix reported to be biodegradable under appropriate environmental conditions represent meaningful steps toward more sustainable OMIEC materials. These advances provide a foundation for the future development of environmentally improved organic bioelectronic devices.

## 2. Materials and methods

### 2.1. Materials

PSS-free PEDOT colloids were synthesized and characterized previously.^23^ From the various colloids dispersions reported, we have selected the PEDOT:NO_3_/PVA (previously denoted PEDOT/PVAL/2) dispersion for this study, as it exhibited the highest electrical conductivity (0.95 ± 0.07 S cm^−1^) and formed continuous, grain-like thin films with well-defined PEDOT-rich particles embedded in a polymer matrix under drop-casting, as evidenced by SEM and AFM in the original work. The colloid was prepared *via* a one-step oxidative polymerization of 3,4-ethylenedioxythiophene (97% purity EDOT, 0.2 M) in an aqueous solution containing 2 wt% of poly (vinyl alcohol) PVA (*M*_w_ = ∼61 000 g mol^−1^) as steric stabilizer. Iron(iii) nitrate nonahydrate (0.5 M) was utilized as oxidant, maintaining a monomer-to-oxidant molar ratio of 1 : 2.5. Therefore, the counterions for the partially oxidized PEDOT shall be nitrate anions, as shown in the XPS results below. After a two-week polymerization period at room temperature, the dispersion was purified by dialysis against 0.05 M nitric acid for seven days. A dialysis membrane with a molecular weight cut-off (MWCO) of 14 000 g mol^−1^ was used to remove residual EDOT monomer, oxidant species, and low-molecular-weight byproducts.^23^ Detailed synthesis approach, material characterization (by UV-vis and Raman spectroscopy) and morphological analysis (by atomic force microscopy and transmission electron microscopy) of the PEDOT colloid dispersion have been reported in our previous work.^23^

Transmission electron microscope (TEM, FEI Tecnai G2 Spirit) was used to characterize the dispersed PEDOT:NO_3_/PVA colloidal particles without additives. The colloids were diluted by a factor of 1000 with 0.05 M nitric acid. TEM reveals the particle size of PEDOT:NO_3_/PVA colloid with an average diameter of 245 ± 44 nm.

Commercial poly(3,4-ethylenedioxythiophene): poly(styrenesulfonate) (PEDOT:PSS, Clevios™ PH 1000, Heraeus Electronics GmbH (Hanau, Germany)) was employed as the reference material. Before processing, the PEDOT:PSS dispersion was filtered using a PES syringe filter with a pore size of 0.45 µm. To prepare the active layers for OECT fabrication, film-forming dispersions were formulated by mixing mixing 94.5% (v/v) of the respective PEDOT-based dispersions (PEDOT:PSS, or PEDOT:NO_3_/PVA) with 0.5% (v/v) 4-dodecylbenezenesulfonic acid (DBSA, Fluka), 5% (v/v) glycerol (Carl Roth). Subsequently, 1% (v/v) (3-glycidyloxypropyl) trimethoxysilane (GOPS, Grolman) was added to the total volume of the previously prepared mixture and stirred for 48 hours. Throughout this work, we refer to the channel material as PEDOT:NO_3_/PVA to explicitly indicate the nitrate-doped PEDOT phase stabilized in the PVA matrix.

### 2.2. Thin-film materials characterization

For the material characterization, glass substrates (1.5 cm × 1.5 cm) and ITO-coated glass substrates (1.5 cm × 1.5 cm) were sequentially cleaned in an ultrasonic bath with a 2% (v/v) aqueous solution of Hellmanex III, deionized water, acetone, and isopropanol, each for 20 min at 50 °C. Afterwards, the substrates were blow-dried using a nitrogen gas stream and prior to usage subjected to oxygen plasma treatment (75 W, 5 min) in a Plasma Etch PE-24 plasma cleaner. For the preparation of composite films used in scanning electron microscopy (SEM) and conductivity measurements, dispersions consisting of 94.5% (v/v) PEDOT:NO_3_/PVA or PEDOT:PSS, 0.5% (v/v) (DBSA), and 5% (v/v) glycerol were prepared, followed by the addition of 1% (v/v) GOPS to the total volume of the mixture. The resulting composite dispersions were deposited by drop-casting onto glass and ITO-coated glass substrates and dried at 120 °C for 30 min for conductivity measurements and SEM characterization, respectively.

The electrical conductivity of the drop-cast or spin-coated films was measured on 1.5 × 1.5 cm glass substrates using the four-point probe station (Ossila), equipped with supplied software for data collection and analysis. Sheet resistance and conductivity were calculated with the Ossila software, which includes the geometric factor correction.

X-ray photoelectron spectroscopy (XPS) measurements were carried out using a Theta Probe XPS-system (Thermo Fisher, GBR), using the Al-Kα X-rays (*hν* = 1486.6 eV) of a non-monochromated twin anode. A 1 mm diameter X-ray spot was utilized for all measurements. High-resolution (HR) spectra were acquired using a hemispherical analyzer with a pass energy of 40 eV and an energy step size of 0.05 eV. All measurements were performed by using a dual flood gun for charging compensation, emitting beams of low energetic electrons (0.5 eV) and a low energetic Ar-ions. Device control, data acquisition and processing were conducted by a software package called Avantage, provided by the system manufacturer. For XPS measurements, the as prepared PEDOT:NO_3_/PVA colloid was used without further additives, and samples were prepared by drop-casting the dispersion onto glass substrates.

Field emission – scanning electron microscopy (FE-SEM) images of PEDOT colloids (at 15 000× and 50 000× magnification) were recorded using a FEI FEG-Quanta 450 instrument (Field Electron and IonCompany, Hillsboro, OR, USA) at the University of Pisa (CISUP). The materials were drop-cast on ITO glass from additives based colloidal solutions. Prior to the analysis, the sample was sputtered with platinum to improve the image quality.

Fourier-transform infrared (FTIR) spectroscopy was carried out using a Bruker Vertex 80 FTIR spectrometer equipped with a Bruker Platinum attenuated total reflectance (ATR) unit. The spectra were recorded with a resolution of 1 cm^−1^ and averaging 200 scans. Samples for FTIR analysis were prepared by drop-casting PEDOT:NO_3_/PVA colloid films onto glass substrates, followed by drying at 100 °C for 30 min and subsequently at 120 °C for 20 min. The dried films were then peeled from the glass substrates, ground into powder, and analyzed by ATR-FTIR.

The film thicknesses were measured using a Bruker Dektak XT profilometer operated with a stylus load of 2 mg.

### 2.3. OECT device fabrication and characterization

OECTs were fabricated on pre-cleaned glass substrates, patterned with Cr/Au source and drain electrodes (S/D) with the channel length *L* = 60 µm and width *W* = 2 mm. The substrates were fixed onto a 200 µm-shadow mask to minimize shadowing effects during metal deposition. Next, a 10 nm chromium (Cr) layer followed by a 100 nm gold (Au) layer was deposited sequentially by thermal evaporation at a pressure of approx. 1–5 × 10^−7^ mbar. In this configuration, the thin Cr interlayer was used to improve the adhesion of the Au-electrodes to the glass substrate. The channel geometries were verified and measured from optical microscope images using KLONK Image Measurement software as shown in the representative micrographs provided in the SI (Fig. S2). Afterwards, the patterned electrode structures were subjected to oxygen plasma treatment (75 W, 5 min) in a Plasma Etch PE-24 plasma cleaner. The active channel layers were then formed by depositing the respective PEDOT-based dispersions containing the aforementioned additives onto the substrates. The PEDOT:PSS films were prepared by spin-coating at 900 rpm for 40 s followed by 1200 rpm for 10 s, whereas PEDOT:NO_3_/PVA films were deposited by drop-casting. Subsequently, the areas surrounding the channel and the contact pads were carefully cleaned with water-wetted cotton swabs. PEDOT:PSS-based devices were annealed at 120 °C for 30 min, while for the drop-cast PEDOT:NO_3_/PVA devices, the films were first dried at 100 °C for 30 min and then subjected to a final annealing step at 120 °C for 20 min. The device fabrication was finalized by fixing a laser-cut polymer well of 3M^TM^ VHB^TM^ Tape consisting of acrylic adhesive with a conformable acrylic foam core on the substrates to confine the aqueous electrolyte. For electrical characterization, 20 µL of phosphate-buffered saline (PBS, 50 mM, pH 7.4) was employed as the electrolyte, and a commercial silver/silver chloride (Ag/AgCl/3M KCl) electrode was served as a non-polarizable gate. All steady-state current–voltage (*I*–*V*) characteristics of the devices were measured under ambient conditions with an Agilent E5273A source-measure unit. The channel geometries of the devices were examined by capturing microscope images with a Nikon Eclipse LV100 ND microscope.

## 3. Results and discussion

### 3.1. Material and thin-film characterization

**Fig. 2:**
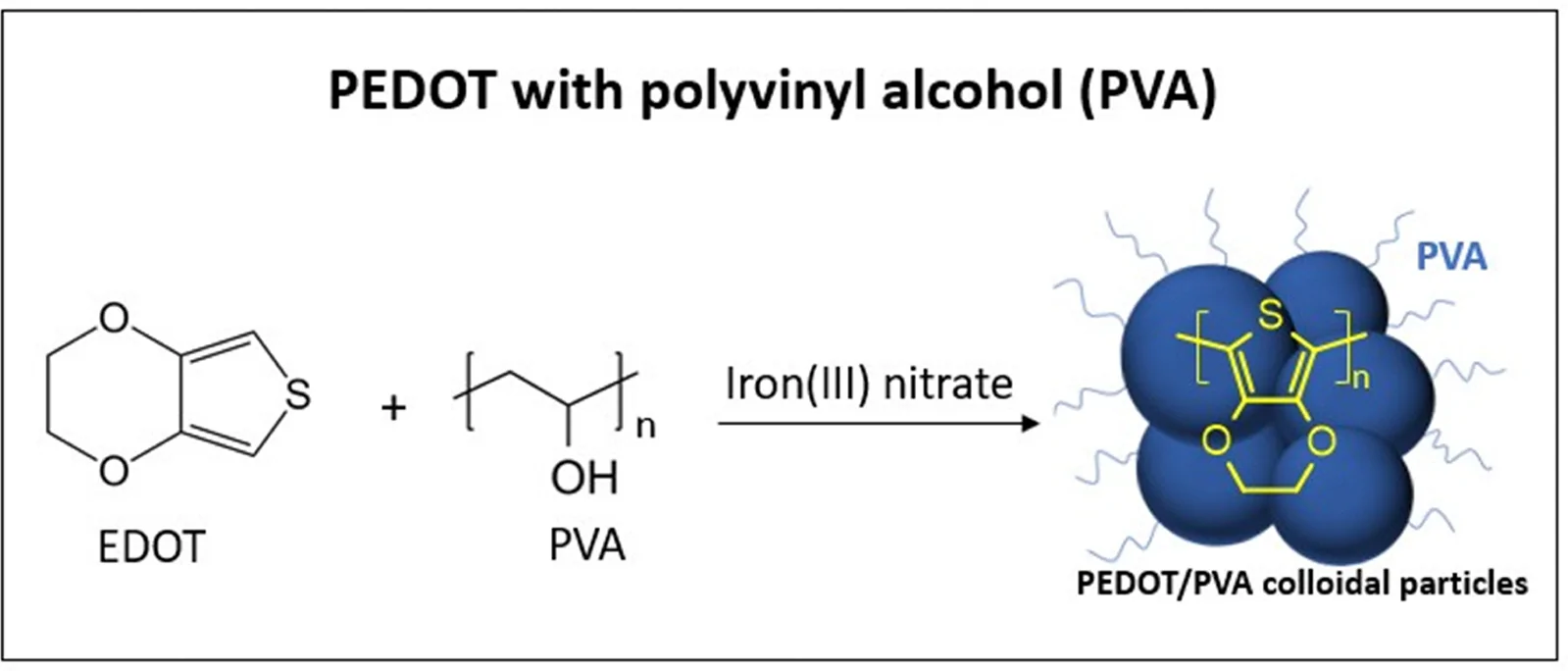
PEDOT colloid with poly(vinyl alcohol) (PVA).

PEDOT colloidal dispersion was prepared by the *in situ* oxidative chemical polymerization of EDOT with iron(iii) nitrate oxidant, in the presence of 2 wt% of PVA as we previously reported.^23^ The preparation of the PEDOT/PVA colloidal dispersion is schematically illustrated in Fig. 2. PVA synthetic biocompatible polymer was involved as a steric stabilizer during oxidation of EDOT monomers to prevent PEDOT particles aggregation by creating a protective stabilizing shell around them. These stabilizing shells reduce van der Waals attraction forces between particles, lower the interfacial energy with the solvent, and introduce steric or electrostatic repulsion to enhance long-term dispersion stability.^40^ In this system, nitrate (NO_3_^−^) acts as the primary counterion, while PVA serves as neutral polymer matrix and film-forming additive, enabling the fabrication of PSS-free PEDOT-based organic mixed conductor. Moreover, nitrate anions play a critical role in the molecular arrangement and conductivity. Detailed material characterization and spectroscopy analysis were performed by UV-vis, Raman and published previously.^23^

**Fig. 3:**
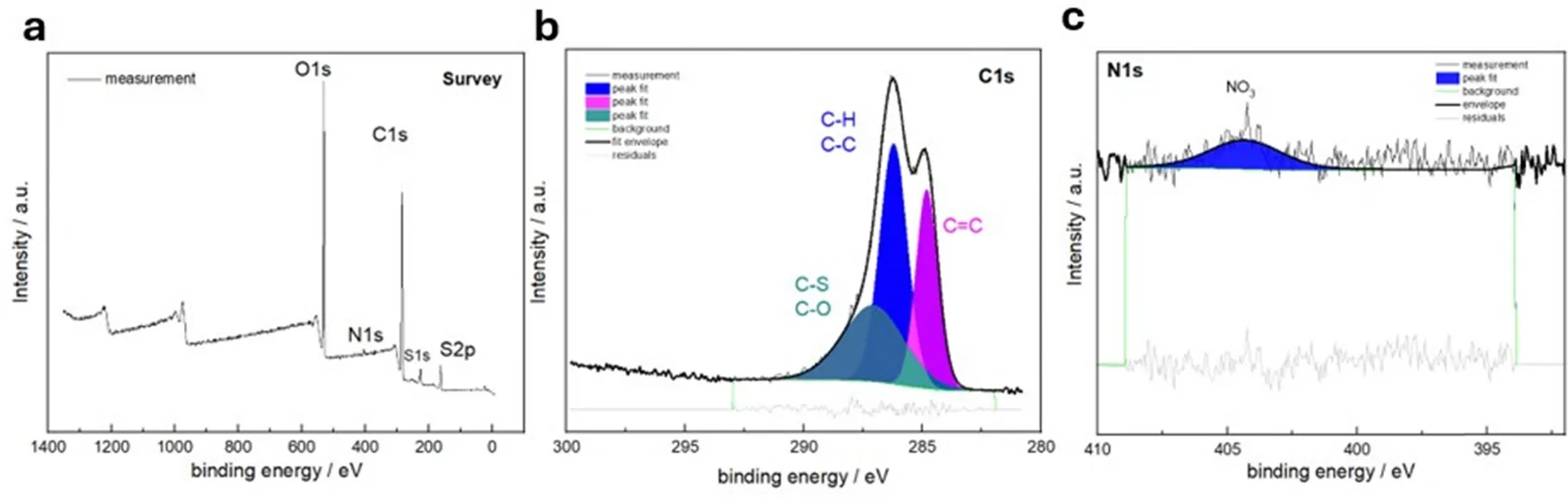
(a) XPS survey spectrum of PEDOT:NO_3_/PVA with the respective high-resolution scan, for (b) C1s and (c) N1s XPS signals.

To further identify the molecular structure of the composite materials, we additionally conducted X-ray photoelectron spectroscopy (XPS) measurements. XPS analysis shown in Fig. 3 reveals the elemental composition of the PEDOT:NO_3_/PVA dispersion film drop-cast on glass substrates. The XPS survey spectra confirms the presence of C, O, N and S as the main elements of the composite.^41^ The deconvolution of C1s high-resolution spectra reveals main component at 284.8 eV for PEDOT:NO_3_/PVA, which are attributed to C–C, C–H and C=C bonds in the aromatic thiophene rings of PEDOT and in the aliphatic chains of the PVA stabilizers, in line with the dominant C–C contribution around 284.8–285.0 eV which are typically observed for PVA-based films and related polymeric systems.^42,43^ Broader contributions at higher binding energy (∼286–287 eV) are assigned to C–O and C–O–C bonds from the oxyethylene units of PEDOT and from hydroxyl (PVA), in line with C 1s deconvolutions of PVA-based nanocomposites and PEDOT/polymer blends that locate C–O, C=O and O–C=O peaks between 286 and 289 eV.^42,43^

The binding energies and chemical assignments of the C1s and O1s spectra are therefore in close agreement with detailed XPS studies on PVA films reported earlier XPS spectra that show C–C/C–H around 285 eV, C–O/C–N at ∼286 eV, C=O at ∼289 eV.^42,44^

The N1s spectrum of PEDOT:NO_3_/PVA displays a clear peak at ≈404.3 eV (Fig. 3c). This binding energy is related to characteristic of nitrate (NO_3_^−^) species. This assignment is supported by XPS measurements of a polyethylene glycol (PEG)/KNO_3_ reference film prepared under identical conditions, which exhibits an N1s component at the same binding energy (Fig. S1c). Therefore, we attribute the nitrogen signal to nitrate dopant anions incorporated in PEDOT:NO_3_/PVA during oxidative polymerization. The S2p spectrum of PEDOT:NO_3_/PVA, shown in Fig. S1d (SI) exhibits a well-defined S2p_3/2_–S2p_1/2_ spin-split doublet with S2p_3/2_ at 163.8/165.1 eV and S2p_1/2_ at ∼164.4/165.6 eV, in good agreement with S2p values reported for PEDOT:PSS and PEDOT:NO_3_/PVA composites. The overall findings demonstrate the successful in-situ polymerization of EDOT in the presence of PVA. A complete elemental analysis with the detailed peak values and calculated atomic ratios of C, O, S and N is presented in Table S1 (SI). The obtained atomic percentages are consistent with the expected chemical composition of PEDOT-to-stabilizer and show no evidence of residual Fe^2+^ ions from the oxidant, in line with previous observations for thoroughly purified PEDOT dispersions.^23,31^ In addition to XPS, ATR-FTIR spectroscopy was employed as a complementary technique to confirm the presence of the characteristic functional groups of both PEDOT and PVA in the composite. The FTIR spectrum of PEDOT:NO_3_/PVA (Fig. S3) further confirms the successful formation of the composite. The characteristic PEDOT bands at approximately 1510–1500 cm^−1^ (C=C stretching), 1320–1280 cm^−1^ (inter-ring C–C stretching), and 1200–1130 cm^−1^ (C–O–C stretching of the ethylenedioxy groups) are clearly observed. The strong absorptions in the 1100–900 cm^−1^ region arise from overlapping C–O vibrations of PVA and PEDOT, whereas the bands below 1000 cm^−1^ are consistent with C–S stretching and deformation vibrations of the PEDOT thiophene ring. These characteristic vibrations are in good agreement with previously reported PEDOT/PVA composite materials, further supporting the coexistence of both PEDOT and PVA in the composite structure.^45–47^ The spectrum exhibits a broad absorption envelope in the approximately 3600–2800 cm^−1^ region. In doped PEDOT, the broad electronic absorption associated with delocalized charge carriers overlaps with the O–H stretching vibrations of PVA, making the hydroxyl stretching band difficult to distinguish.

**Fig. 4:**
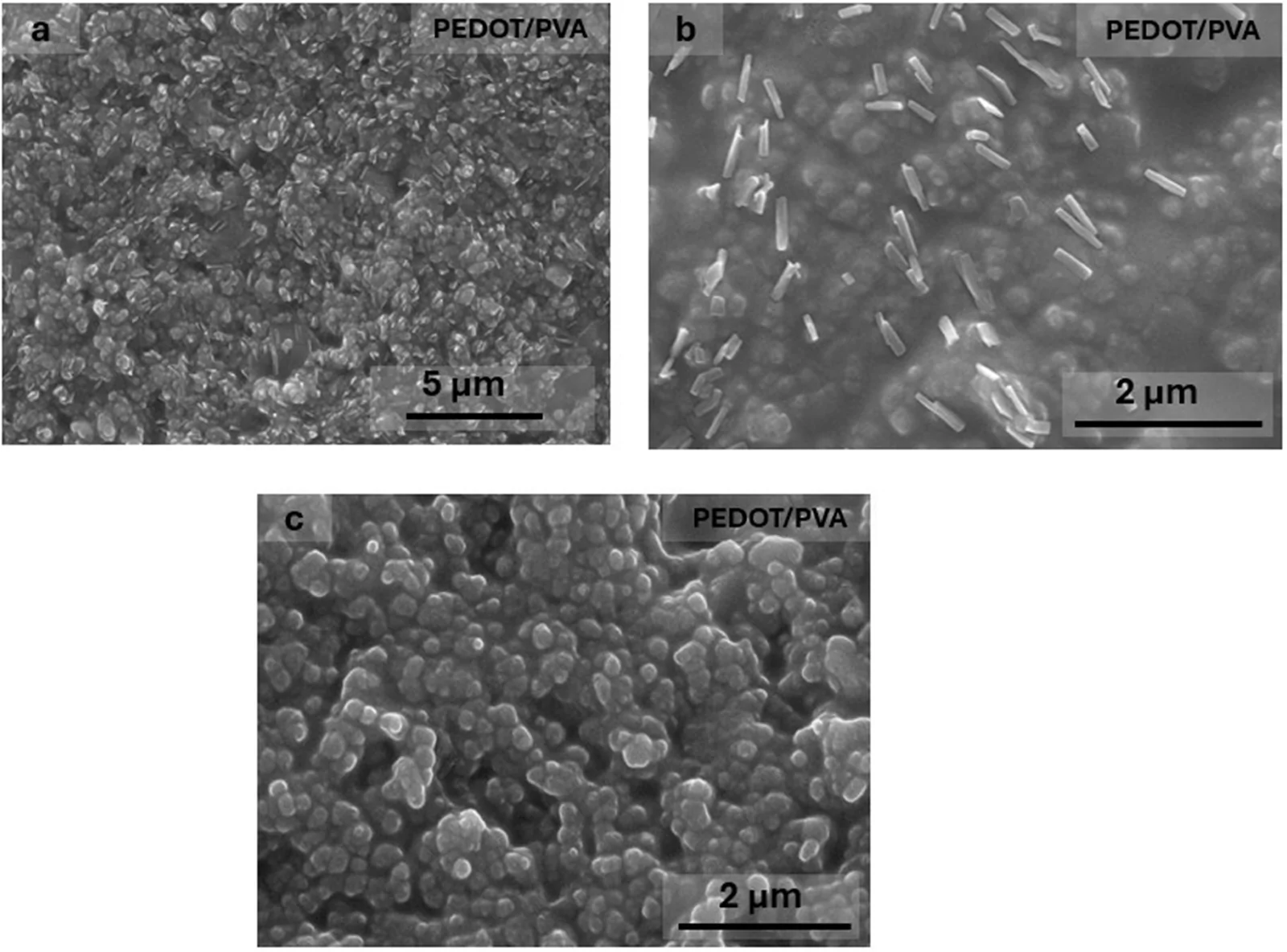
SEM images of PEDOT:NO_3_/PVA-based colloidal film: (a) at a lower magnification of 15 000× (scale bar = 5 µm), and with the higher magnification of 50 000× for (b) a region containing rod-like crystalline domains and (c) a region without visible crystalline features, (scale bars= 2 µm).

Scanning electron microscopy (SEM) was used to investigate the surface morphology of the PEDOT:NO_3_/PVA film prepared as OECT channel layer from the colloidal dispersion containing DBSA, glycerol and GOPS as standard additives for OECTs.^48^ In this case, drop-casting of the material onto ITO-coated substrates yielded continuous coatings without macroscopic cracks or pinholes, as depicted in Fig. 4. The surface morphology consists of densely packed globular features forming a continues polymer network as shown in Fig. 4a and c, consistent with previously reported observations on additive-free PEDOT colloid films.^23^ The PEDOT:NO_3_/PVA films exhibit compact, spherical grains with submicrometer lateral size, indicating efficient coalescence of PEDOT phase in the presence of PVA. This interpretation is further supported by the TEM images (Fig. S4), which show a core–shell morphology, where the higher-electron-density core is attributed to PEDOT and the surrounding lower-electron-density shell to the PVA stabilizer. In addition, Fig. 4a and b show individual rectangular, rod-like crystalline features distributed throughout the film, which are consistent with crystalline PVA domains reported in the literature.^49^ A similar morphology has been reported for PEDOT:PSS/PVA blends when PVA is employed as a polymer matrix and crosslinking agent.^50^ The XPS and FTIR analyses confirm the coexistence of PEDOT and PVA within the composite, while the TEM images provide structural evidence for a PEDOT core–PVA shell architecture, supporting the proposed interpretation of the observed SEM morphology. The incorporation of DBSA, glycerol and GOPS leads to smoother and more continuous films than the corresponding additive-free colloids while preserving the characteristic morphology of PEDOT:NO_3_/PVA system.^23^

The electrical conductivity of the corresponding PEDOT:NO_3_/PVA colloid-based thin film was characterized and summarized in Table 1. After one-year of storage period, the colloidal dispersion was mixed with the additives DBSA, glycerol, and GOPS according to the same formulation employed for OECT fabrication, and drop-cast onto glass substrates. The resulting films exhibited a moderate decrease in conductivity compared to the additive-free layers reported previously of about 0.95 ± 0.07 S cm^−1^.^23^ In the present study, the conductivity obtained for the additive-containing film was 0.56 ± 0.14 S cm^−1^. This moderate loss in conductivity can be attributed, on the one hand, to changes in the colloids during long-term storage (≈260 days), which are known to promote aggregation and evolution of particle size and polydispersity in these systems, as reported in our previous work,^23^ and, on the other hand, to the incorporation of GOPS as a crosslinking agent. GOPS is widely used to improve the water stability and adhesion of PEDOT-based films, but it is also known to alter film morphology and reduce electrical conductivity by introducing insulating crosslinked regions.^48^ However, the conductivity measured here remains within the same order of magnitude as the originally reported value, indicating that the colloidal PEDOT network largely preserves its electronic transport capability even after long-term storage and with the three-additive formulation.

**Table 1:** Overview of OECT device performance metrics and measured conductivity of the respective polymer colloids thin-films

Material	Film conductivity^a^/S cm^−1^	*g*_m_/mS	*g*_m,norm_/S cm^−1^	On/off ratio	*N* b
PEDOT:NO_3_/PVA	0.56 ± 0.14	7.79 ± 2.12	0.16 ± 0.04	77 ± 50	4
PEDOT:PSS	289.9 ± 29	15.60 ± 1.52	13.42 ± 2.1	188 ± 169	3

a Film conductivity values were obtained for PEDOT:NO_3_/PVA and PEDOT:PSS dispersion from 2 independent substrates, with three four-probe measurements per substrate (total *n* = 6).

b *N* denotes the number of OECT devices used to extract the steady-state transfer parameters.

### 3.2. OECT device characterization

**Fig. 5:**
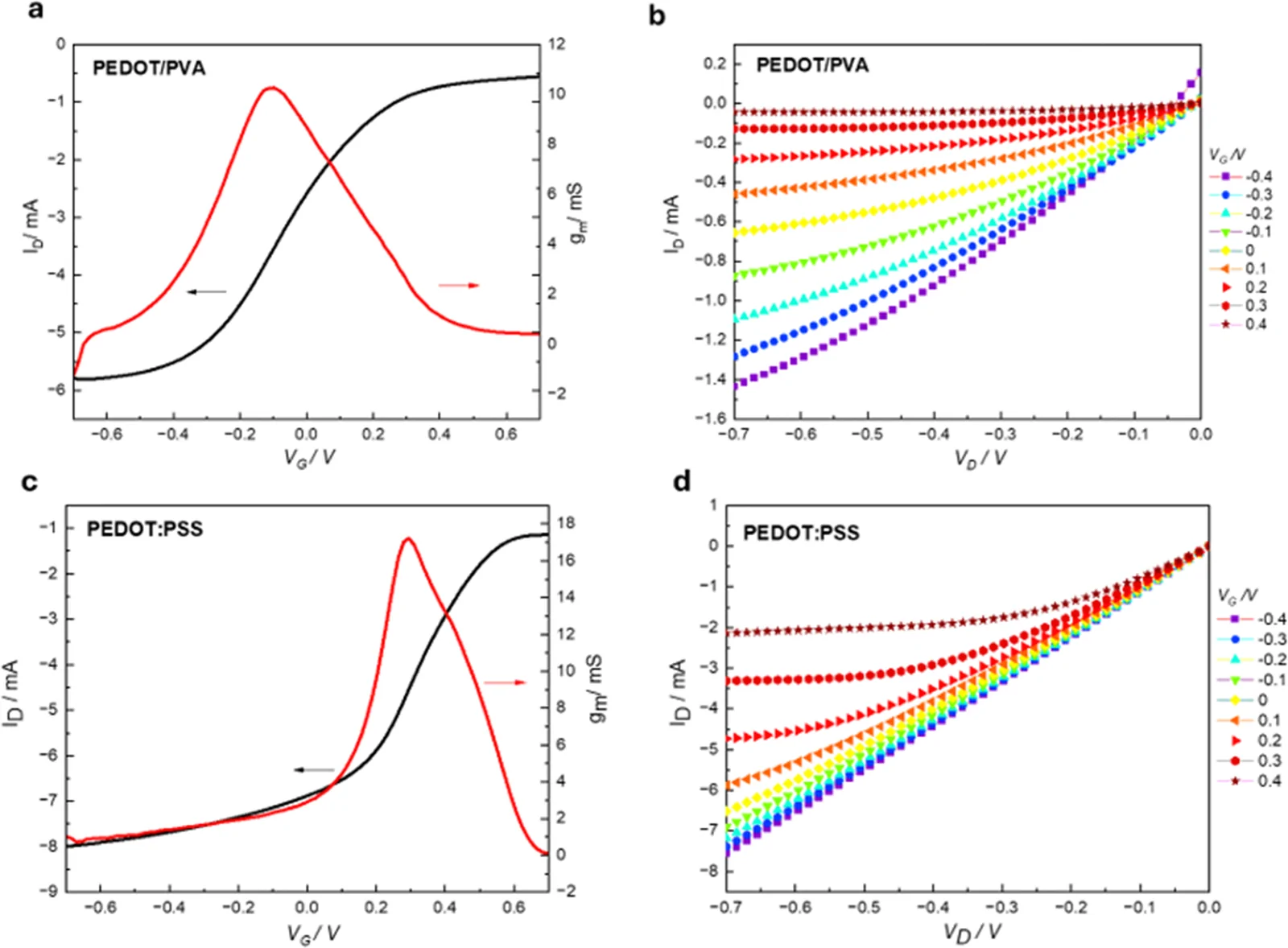
Steady-state electrical characterization of PEDOT:NO_3_/PVA (*W* = 2.1 mm, *L* = 62.5 µm, *d* = ∼16.5 µm), and PEDOT:PSS-based micro-OECTs (*W* = 2.1 mm, *L* = 35.1 µm, *d* = ∼0.2 µm). (a) and (c), Transfer characteristics and the corresponding transconductance curve (at applied constant drain voltage, *V*_D_ = −0.7 V) and (b) and (d) the corresponding output characteristics, respectively. Scan rate = 15 mV s^−1^.

To evaluate the ion-to-electron transduction capability of PEDOT:NO_3_/PVA dispersion for bioelectronic applications, micro-OECTs were fabricated comprising drop-cast PEDOT:NO_3_/PVA film as the active channel. Fig. 5a represent the steady-state transfer (*I*_D_*vs. V*_G_) for the best-performing exemplary device. The data was recorded at a constant drain voltage *V*_D_ = −0.7 V, and the gate voltage (*V*_G_) was swept from −0.7 to +0.7 V. The corresponding output curves (*I*_D_*vs. V*_D_), shown in Fig. 5b, were obtained by sweeping *V*_D_ from 0 to −0.7 V while varying *V*_G_ between −0.4 and +0.4 V. The output characteristics of the PEDOT:NO_3_/PVA OECTs, recorded at fixed *V*_*G*_, show a monotonic and nearly linear increase |*I*_D_| of with |*V*_D_| over the investigated bias range, which is consistent with the typical behavior reported for PEDOT-based OECTs.^51^

The transfer characteristic show that the drain current decreases significantly as *V*_G_ increases towards more positive values. The observed p-type OECTs were measured in depletion mode, where at zero gate bias the PEDOT channel is in a highly conductive “ON” state, and a positive *V*_G_ drives cations from the electrolyte into the channel, compensating the fixed anionic dopants and extracting holes from PEDOT, thereby reducing its conductivity and eventually approaching an “OFF” state.^13,31,52^ When *V*_G_ is biased negatively beyond the turn-on voltage, anions penetrate the channel, leading to increased hole density and restoring the high-conductance “ON” state.^13,52^ This is fully consistent with the established depletion-mode operation of PEDOT-based OMIECs. The transconductance (*g*_m_), a key figure of merit for OECTs, represents the efficiency for ion-to-electron transduction.^31^ It is defined as the slope of the transfer curve and is plotted (red curve) alongside the transfer characteristics (black curve) in Fig. 5a, *g*_m_ = ∂*I*_D_/∂*V*_G_, Table 1 summarizes the statistical analysis of the maximum transconductance (*g*_m_) and device performance metrics for the different OECTs based on PEDOT dispersions.

To enable a fair comparison of device performance across different geometries and channel thickness, we calculated the normalized maximum transconductance, *g*_m,norm_, according to the following equation:
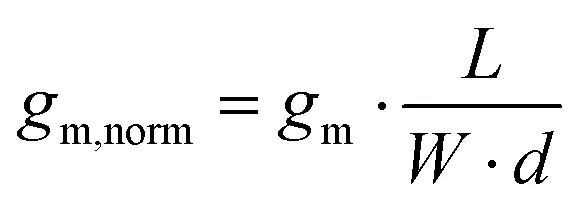
where *L*, *W*, and *d* denote channel length, width, and thickness, respectively. This normalization removes trivial dependencies on channel geometry and active-layer thickness and isolates the intrinsic mixed ionic–electronic transport capability of PEDOT:NO_3_/PVA dispersion. The resulting OECTs exhibited average *g*_m_ values of 7.79 ± 2.1 mS (*N* = 4), with the best device performance reaching *g*_m_ = 10.27 mS. The corresponding normalized transconductance was *g*_m,norm_ = 0.16 ± 0.04 S cm^−1^, for devices with average dimensions *L* = 57.0 ± 8.0 µm, *W* = 2123 ± 11 µm and *d* = 13.4 ± 2.1 µm. These values place the present devices among the highest-performing PVA-based OECT active channels summarized in Table 2. For instance, crosslinked PVA/PEDOT:PSS conducting hydrogels reported by Gregorio *et al.* exhibit a maximum transconductance of about 1.05 µS for 12 µm drop-cast films, corresponding to normalized *g*_m_ of 0.001 mS cm^−1^, reflecting the diffusion-limited mixed transport in swollen insulating matrices.^38^ Likewise, PVA-modified PEDOT:PSS inks developed for fully printed 3D-OECTs demonstrated *g*_m_ values around 5 mS, corresponding to normalized *g*_max_ of 8.7 mS cm^−1^ where PVA primarily improves printability and mechanical stability rather than charge transport.^22^ A comparable dependence on PVA concentration has been reported in PEDOT:PSS/PVA-based OECTs where average *g*_m_ values in the range *g*_m_ ∼0.3–1.5 mS, corresponding to normalized *g*_m_ of 0.3 S cm^−1^ were obtained. In their study, PVA was blended directly into PEDOT:PSS, followed by sulfuric acid post-treatment.^39^ Similar to our system, PVA acts as a neutral polymer matrix that increases the insulating phase and disrupts the PEDOT percolation pathway, which leads to a progressive decrease in both transconductance and volumetric capacitance. A further illustrative example is provided by CX-PVA/CX-P3HT neuromorphic OECTs, where the introduction of a crosslinked PVA interlayer increased the transconductance from 0.01 to 1.41 mS by improving ion transport, while still remaining in the low-to-medium *g*_m_ regime compared with PEDOT-based channels.^53^ Importantly, the vast majority of PVA-based OECT systems reported to date are based on PEDOT:PSS, where the polystyrene sulfonate (PSS) serves as both dispersant and counterion.^22,38,53^ In such architectures, PVA is typically incorporated as a matrix, crosslinker, blend component, or processing additive within the channel. By contrast, the devices presented here rely on PSS-free PEDOT:NO_3_/PVA colloids, in which PVA functions exclusively as a steric stabilizer rather than as an ionic polyelectrolyte matrix. The chemical structure of PVA further provides an explanation for the microstructural and charge transport behavior observed in PEDOT:NO_3_/PVA films. The hydroxyl groups of PVA may interact with the PEDOT backbone *via* hydrogen bonding or secondary polar interactions. Such interactions can enhance film cohesion and potentially reduce excessive dedoping by restricting ion mobility within the channel.

**Table 2:** Poly(vinyl alcohol)-based organic electrochemical transistors

Material	Device component	Study purpose/PVA role	*g*_m_/mS	*g*_m,norm_/S cm^−1^	*L*/µm	*W*/µm	*d*/µm	Ref.
PVA/gelatine/borax electrolyte	Solid electrolyte	Solid state PEDOT:PSS-based OECT	54	108	200	1000	1–2	14
PVA/PEDOT:PSS hydrogels	Channel material	OECT-crosslinker	0.001	0	20	100	20	38
PEDOT:PSS–PVA ink	Channel material	3D-printed OECT	∼5	8.7	70	200	2	22
PEDOT:PSS–PVA	Channel material	Crosslinker	0.3–1.5	0.3	20	80	16–58	39
CX-PVA/CX-P3HT	Channel material	Neuromorphic OECT	0.01–1.4					53
PEDOT:NO_3_/PVA	Channel material	Matrix for PSS-free PEDOT	10.2	0.18	57	2123	13.4	This Work*

PEDOT:PSS, the most widely used OMIEC material in OECTs. In our study, spin-coated PEDOT:PSS thin layers with the thickness of ∼268 nm were employed as the reference channels for comparison. The best-performing PEDOT:PSS-based device (in Fig. 5c and d) with a similar S/D geometry, exhibited average *g*_max_ of 15.6 ± 1.5 mS and a normalized transconductance of about 13.4 ± 2.1 S cm^−1^. These values fall within the typical range reported for high-performance PEDOT:PSS-based OECTs, including both early benchmark devices^54^ and more recent architectures in which aqueous stability and electronic performance are improved either by using GOPS as a substrate-bound adhesion layer instead of blending it into the channel^55^ or by applying short high-temperature annealing of GOPS-free PEDOT:PSS films.^56^

Compared to the optimized PEDOT:PSS reference devices, the PEDOT:NO_3_/PVA channels discussed above exhibited *g*_m,norm_ = 0.16 ± 0.04 S cm^−1^, approximately two orders of magnitude lower normalized transconductance. While the PEDOT:NO_3_/PVA films exhibit lower electronic conductivity and normalized transconductance, they offer distinct advantages, including a PSS-free composition, excellent colloidal stability, and the ability to form mechanically robust thick films.

Despite the relatively large channel thickness (14–16 µm), the PEDOT:NO_3_/PVA OECTs demonstrates effective gate modulation, with an average ON/OFF current ratio of 77 ± 50, compared to 188 ± 169 for the PEDOT reference devices. Although the modulation depth is lower than that of PEDOT, the relatively high ON/OFF ratio indicates efficient volumetric electrochemical doping/dedoping throughout the channel. This suggests that the thick channel does not prevent electrolyte penetration or effective gate modulation. Although thinner OECT channels are generally desirable for faster switching, attempts to prepare submicrometer PEDOT:NO_3_/PVA films by spin-coating (Fig. S5) were unsuccessful under the investigated conditions because the colloidal films did not form a continuous conductive network. Consequently, drop-casting (Fig. S5 and Fig. S6) was selected as the most reliable fabrication method for this proof-of-concept study.

## 4. Conclusions

In this study, we investigated the applicability of PEDOT colloidal dispersion stabilized with PVA as organic mixed ionic–electronic conductor (OMIEC) in OECTs. X-ray photoelectron spectroscopy and scanning electron microscopy analyses confirmed the successful in-situ polymerization of EDOT in the presence of PVA and verified the PSS-free nature of the resulting films. Although the incorporation of neutral polymers lowers the initial electrical conductivity due to dilution of the PEDOT phase, it significantly improves the film formation and appears to moderate excessive dedoping under applied bias. OECTs based on PEDOT:NO_3_/PVA films operated in the depletion mode and exhibited transconductance values exceeding 10 mS, comparable to PEDOT:PSS/PVA-based OECTs despite the complete absence of PSS, highlighting that PSS-free PEDOT:NO_3_/PVA can support mixed ionic–electronic transport suitable for bioelectronic applications.

Building on these findings, the electronic conductivity of the PSS-free PEDOT:NO_3_/PVA system could be further enhanced by adopting strategies successfully established for PEDOT:PSS, including secondary doping with polar solvents, mild acid treatments, and post-processing approaches. Such optimization is expected to improve the film microstructure, electrical conductivity, and mixed ionic–electronic transport. Furthermore, inspired by recent reports on GOPS surface functionalization and thermal processing of GOPS-free PEDOT:PSS films, future studies may explore GOPS-free PEDOT:NO_3_/PVA formulations combined with optimised thermal annealing protocols to further enhance device performance and long-term stability. Finally, the soaking stability (*I*_D_ retention after prolonged electrolyte soaking) and switching stability of PEDOT:NO_3_/PVA OECTs should be systematically evaluated for long-term bioelectronic applications.

## Author contributions

D. S. conceptualization, investigation (primary), visualization, writing, – review & editing; I. M. M. investigation, visualization, writing, – review & editing; M. C. investigation, formal analysis (XPS); K. M. methodology, investigation; N. M. funding acquisition; M. C. S. project administration; P. B. supervision; N. S. S. project administration, funding acquisition; S. T. conceptualization, supervision, project administration, funding acquisition, writing – review & editing.

## Conflicts of interest

The authors declare no conflicts of interest.

## Supplementary Material

MA-007-D6MA00785F-s001

## Data Availability

The data supporting this article have been included as part of the supplementary information (SI). Supplementary information is available. See DOI: https://doi.org/10.1039/d6ma00785f.
