# Supplementary material for: Water-processable PSS-free PEDOT:NO3/PVA colloid for sustainable organic electrochemical transistors
Source: Mater Adv. 2026 Aug 14;7(21):10605–15. doi: 10.1039/d6ma00785f (PMC13504250; doi:10.1039/d6ma00785f)
Supplement: Supplementary file 1 [file MA-007-D6MA00785F-s001.pdf]

Supplementary Information (SI) for Materials Advances.
This journal is © The Royal Society of Chemistry 2026

Supplementary Information (SI)

Water-processable PSS-free PEDOT:NO_3/PVA colloid for Sustainable Organic Electrochemical Transistors

Donia Saadi [1,2,3*], Islam Minisy[3], Munise Cobet[2], Katharina Matura[2], Niko Münzenrieder[1], Markus Clark Scharber[2], Patrycja Bober[3], Niyazi Serdar Sariciftci[2], and Serpil Tekoglu[2*]

[1] Faculty of Engineering, Free University of Bozen-Bolzano, Bozen-Bolzano 39100, Italy

[2] Linz Institute for Organic Solar Cells (LIOS) and Institute of Physical Chemistry, Johannes Kepler University Linz, Linz, Austria

[3] Institute of Macromolecular Chemistry, Czech Academy of Sciences, 16200 Prague, Czech Republic

*Corresponding Authors: donia.saadi@unibz.it, serpil.tekoglu@jku.at

Table of Contents

Figures:

Figure S1: X-ray photoelectron spectroscopy analysis.

Figure S2. Representative optical micrographs of the PEDOT:NO_3/PVA OECT channel geometry.

Figure S3. FTIR spectrum of the PEDOT:NO_3/PVA film.

Figure S4. TEM images of PEDOT:NO_3/PVA colloid without additives.

Figure S5. Four-point probe samples and measurement setup.

Figure S6. Representative stylus profilometry data of a drop-cast PEDOT:NO_3/PVA film used for thickness determination.

Tables:

Table S1. XPS atomic percentages for PEDOT:NO_3*/PVA and* PEG/KNO_3 films.

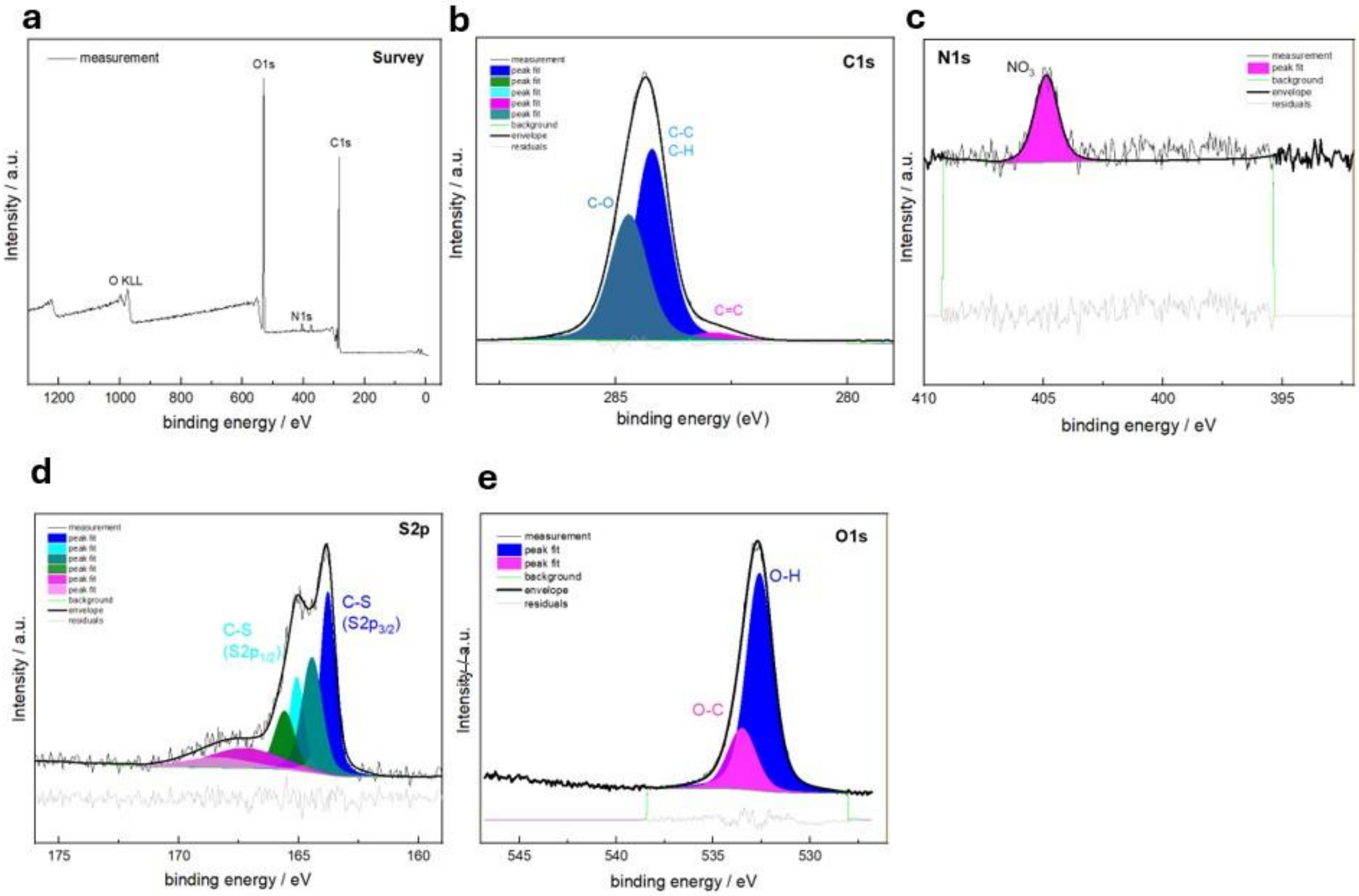

Figure S1. X-ray photoelectron spectroscopy analysis. **a)** Survey spectra of PEG/KNO_3; high-resolution XPS images of **b)** PEG/KNO_3 (C1s); **c)** PEG/KNO_3 (N1s) and **d)** PEDOT/PVA (S2p); **e)** PEDOT/PVA (O1s).

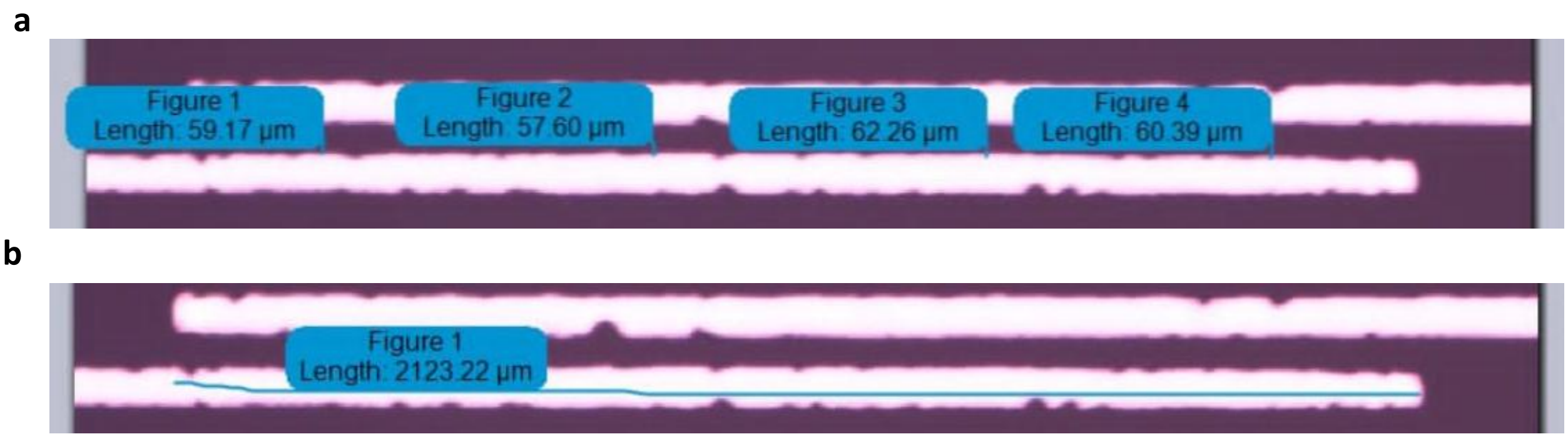

Figure S2. Representative optical micrographs of the PEDOT:NO_3/PVA OECT channel geometry analyzed using KLONK Image Measurement software. **a)** Four independent measurements of the source–drain gap (59.17, 57.60, 62.26, and 60.39 μm) yield an average channel length of L = 60 ± 2 μm. **b)** Determination of the total PEDOT:NO_3/PVA stripe length ($L_{(film)} \approx 2123$ μm) used to calculate the SD overlap ratio $\left(A_{ch}/A_{film} \approx 0.03 \right)$.

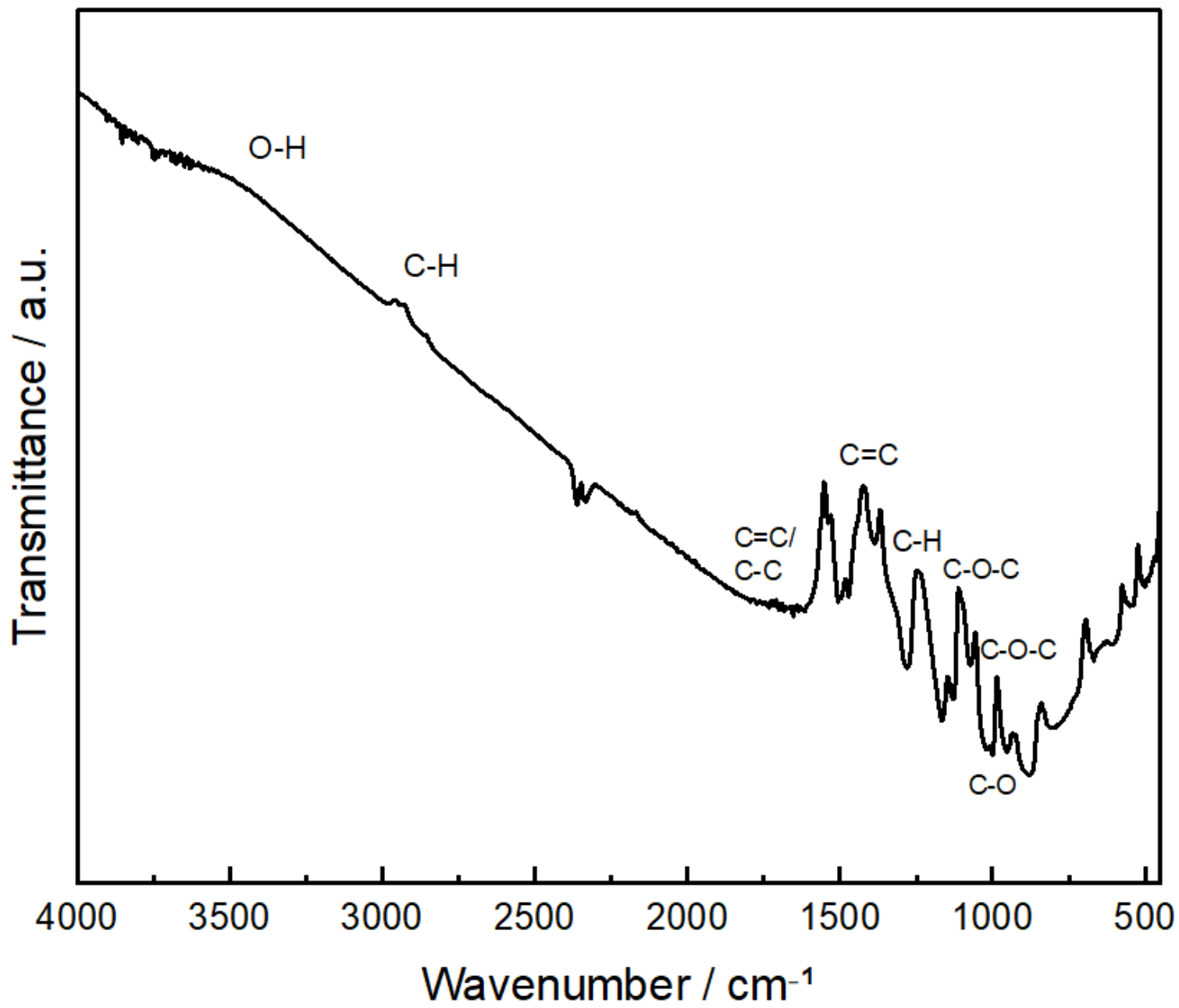

Figure S3. FTIR spectrum of the PEDOT:NO_3/PVA film.

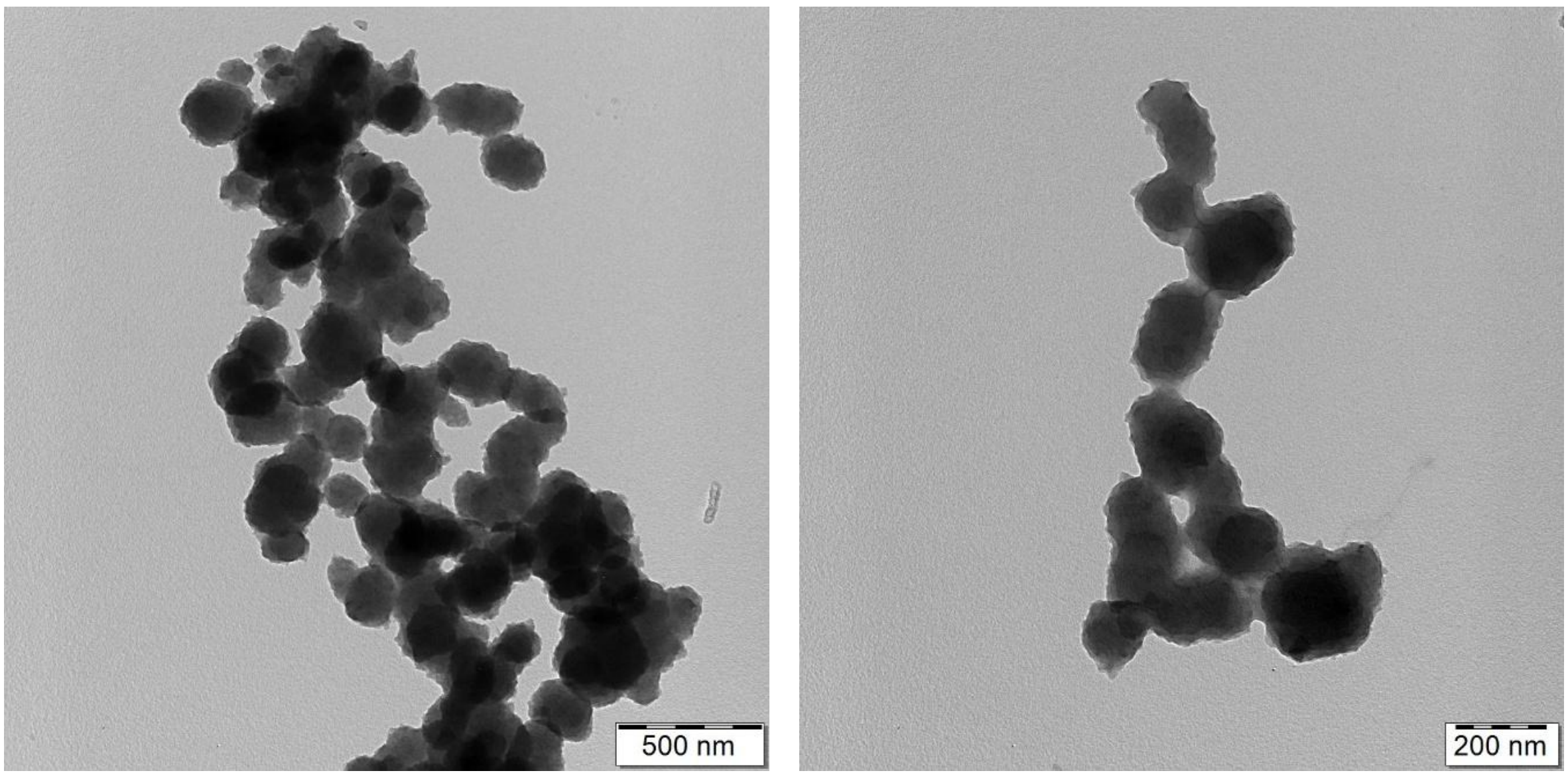

Figure S4. TEM images of PEDOT:NO_3/PVA colloid without additives.

Table S*1*. XPS atomic percentages for PEDOT:NO_3*/PVA and* PEG/KNO_3 films.

Sample	**Name**	**Peak / eV**	**FWHM /eV**	**Area (P) / CPS eV**	**Atomic percentage / %**
PEDOT/PVA	C1s	286.21	1.38	4011.95	27.2
	C1s Scan A	284.8	1.08	2596.3	17.6
	C1s Scan B	287.06	3.04	2804.5	19
	N1s	404.33	3.33	324.75	1.8
	O1s	532.6	1.63	8872.32	22.8
	O1s Scan A	533.51	1.76	2703.54	6.9
	S2p3 Scan A	163.78	0.73	357.42	1.8
	S2p1 Scan A	165.08	0.73	182.57	0
	S2p3 Scan B	164.44	1	310.87	1.6
	S2p1 Scan B	165.59	1	158.79	0
	S2p3 Scan C	167.25	3.45	186.6	0.9
	S2p1 Scan C	168.4	3.45	95.31	0
PEG/KNO_3	C1s	284.22	0.88	4827.64	33.0
	C1s Scan A	290.55	1.01	634.32	4.3
	C1s Scan B	293.4	0.98	316.92	2.1
	C1s Scan C	282.89	1.18	273.83	1.8
	C1s Scan D	284.72	0.97	3509.82	24.0
	N1s	404.88	1.17	348.36	1.48
	O1s	530.33	0.89	2124.24	5.5
	O1s Scan A	530.82	1.15	10600.48	27.5
	C1s	284.22	0.88	4827.64	33

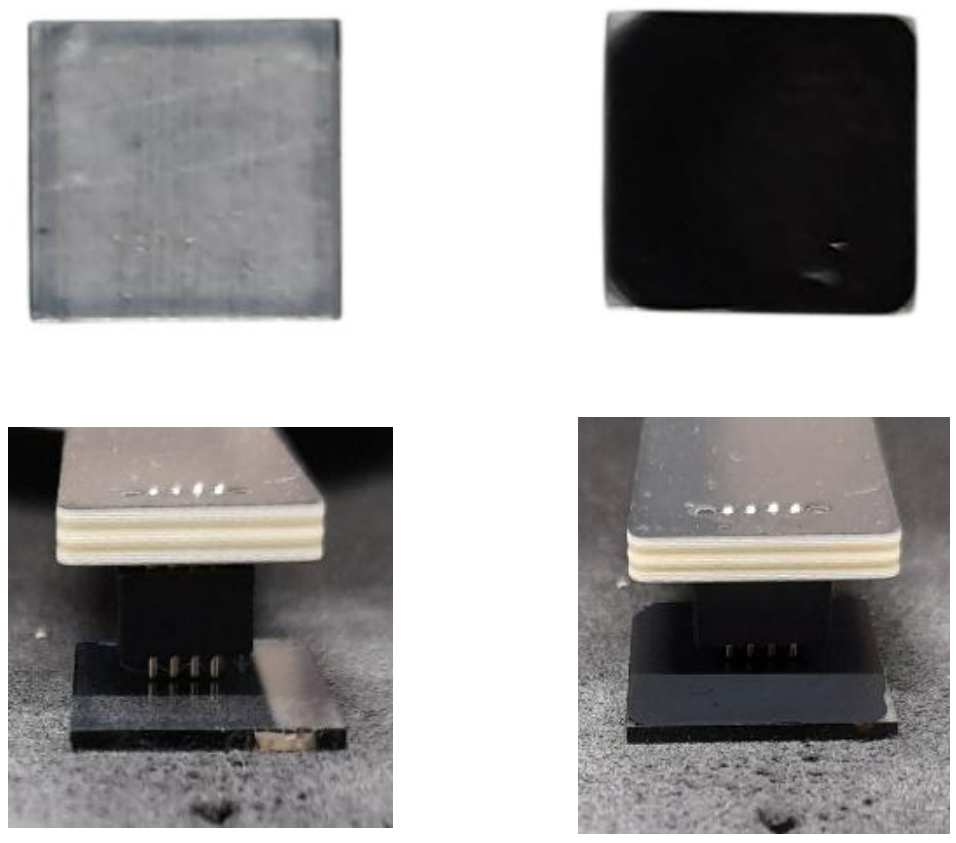

Figure S5. Four-point probe samples and measurement setup. Top: 1.5 × 1.5 cm glass substrate without coating (left) and the same substrate uniformly coated with a PEDOT:NO_3/PVA film (right), as used for conductivity measurements. Bottom: photographs of a coated sample positioned under the Ossila four-point probe head, illustrating the probe geometry and contact configuration during measurement.

As shown in **Figure S5**, the 1.5 × 1.5 cm glass substrates were uniformly coated with PEDOT:PSS (spin-coated) or PEDOT:NO_3/PVA (drop-casted) that were used for the four-point probe experiment, as well as the coated sample positioned under the Ossila four-point probe head during measurement.

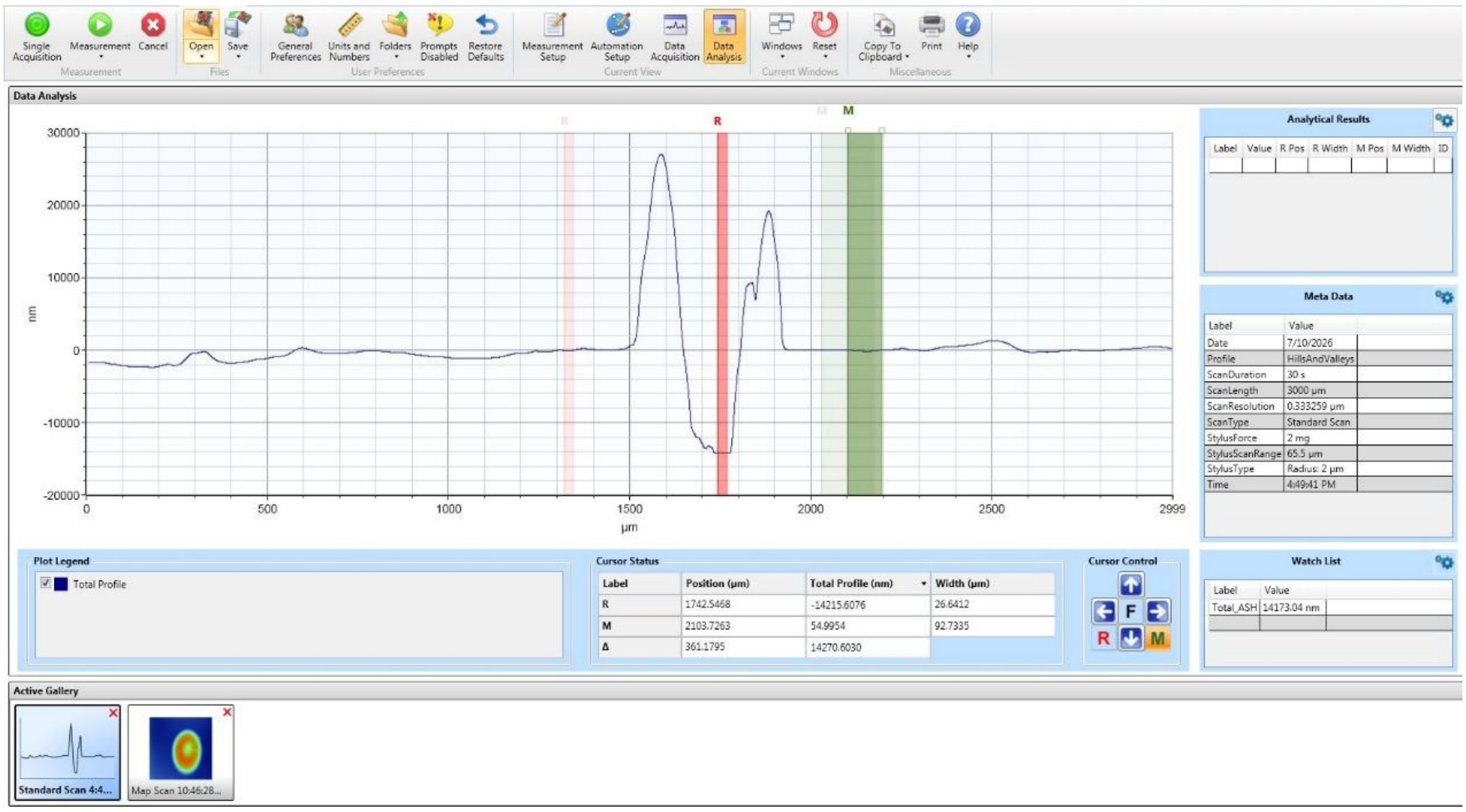

Figure S6. Representative stylus profilometry data of a drop-cast PEDOT:NO_3/PVA film used for thickness determination.

The profilometry data confirm that the drop-cast PEDOT:NO_3/PVA films exhibit local thickness variations and surface undulations, which are characteristic of solvent evaporation and particle redistribution during the drying of colloidal films. The film thickness was determined from the step height measured across deliberately scratched region exposing the underlying glass substrate, yielding a representative average film thickness of approximately **14.3 µm** for the profile shown in **Figure S6**. This value is consistent with the thickness measured for those specific channels used in characterization.

To account for sample-to-sample variations, the thickness was **measured individually for each device** rather than assigning a single nominal value to all films. The corresponding measured thickness was then used in the calculation of the conductivity and normalized transconductance $g_{m,norm}$. Therefore, the reported $g_{m,norm}$ values should be interpreted as geometry-normalized estimates based on the measured average thickness. Any local thickness variations contribute to the experimental uncertainty and are reflected in the reported standard deviations.
